# Clinical outcomes of tibial nonunion treatment through the combination of PRP, autogenous iliac bone grafting, and augmentation plating

**DOI:** 10.3389/fsurg.2025.1573679

**Published:** 2025-07-03

**Authors:** Guoliang Lu, Zhiqian Huo, Enliang Chen

**Affiliations:** ^1^ Eighth Clinical Medical College, Guangzhou University of Chinese Medicine, Foshan, Guangdong, China; ^2^ Department of Orthopedics, Foshan Hospital of Traditional Chinese Medicine, Foshan, Guangdong, China

**Keywords:** tibial nonunion, platelet-rich plasma, autologous bone graft, augmentation plating, fracture healing time

## Abstract

**Objective:**

To explore the clinical efficacy of PRP (Platelet-Rich Plasma), autogenous bone combined with augmentation plating in the treatment of tibial nonunion.

**Methods:**

A total of 45 patients with tibial nonunion who fulfilled the inclusion criteria were meticulously selected and subsequently randomized into three distinct groups: Group A, which received PRP, autogenous bone grafting, and augmentation plating; Group B, which underwent autogenous bone grafting and augmentation plating; and Group C, which only received autogenous bone grafting. Each group comprised 15 patients. Detailed records were maintained for gender, age, BMI (Body Mass Index) as general information, surgery duration, blood loss, length of hospital stay, fracture healing time, and the Fernadez-Esteve score at three specific time points.

**Results:**

No statistically significant differences were observed in the general demographic data, including gender, age, and BMI, among the three groups (*P* > 0.05). In terms of bone healing time, Group A exhibited the shortest duration, followed closely by Group B and then Group C. Additionally, Group A demonstrated significantly higher Fernadez-Esteve scores compared to Group B, with Group C trailing behind, at 3, 6, and 9 months postoperatively.

**Conclusion:**

PRP, autogenous bone combined with augmentation plating in the treatment of tibial nonunion can promote fracture healing and repair, improve fracture healing rate, and the clinical effect is significant.

## Introduction

Tibia fractures are the most common long bone injuries encountered in the trauma population (1). Due to the anatomical structure and relatively poor perfusion in the distal third of the leg, fractures in this part of the body—in comparison with other locations—relatively commonly result in disturbed healing and nonunion (2). The incidence of non-union was reported to be between 8% and 13% (3–5), and could reach 30% (6). These fractures impose the healthcare system to tremendous economic burden, as it was reported that mean expense for treatment of non-union of humerus, femur and tibia is 31,132, 34,400 and 32,660 USD, respectively (7).

Therefore, how to treat tibial nonunion has become the research focus of modern trauma orthopedics. The treatment modalities have evolved from exchange nailing and intramedullary nailing to include the utilization of fine wire and hexapod external fixators (8). Among them, the application of augmentation plating has brought a new treatment prospect for nonunion after intramedullary nailing of limbs. This approach uses the load-sharing capacity of the nail with good axial and bending strength, while the plate provides additional rotational control, as it is believed that rotational instability is the main cause for the non-unions of the diaphyseal long bone fractures (9).

Deriving from the multiple centrifugations of the own peripheral blood, platelet-rich plasma includes considerable amounts of growth factors, such as transforming growth factor, plateletderived growth factor, endothelial growth factor, vascular endothelial growth factor, fibroblast growth factor, insulin-like growth factor, which are the major components in the complex bone healing process and could activate and regulate many aspects of osteoblasts, osteoclasts, and stem cells (10, 11). Recently, numerous basic experimental studies have confirmed the potential therapeutic value of PRP in repair of bone and cartilage tissues (12).

The aim of this retrospective case series was to study the clinical efficacy and safety of combined used of PRP, augmentation plating and autogenous bone in patients with nonunion of tibia. As we know, there was no related systematic review published yet.

## Materials and methods

This study received approval from our institution's ethics committee, and all patients provided informed consent prior to their participation. Between January 2022 and January 2024, 45 patients with tibial non-union underwent surgical treatment. The inclusion criteria were as follows: (1) More than nine months had elapsed since intramedullary nailing for tibial shaft fracture; (2) x-ray examination revealed a clearly visible fracture gap, sclerotic fracture ends, absence of continuous bone trabecular formation between the callus, and no signs of callus growth in the preceding three months; (3) No evident fixation failure or fracture of the original internal fixation was noted on x-ray. The exclusion criteria encompassed: (1) Septic non-union(the patient exhibits elevated preoperative infection indicators, including white blood cell count, erythrocyte sedimentation rate, procalcitonin levels, and C-reactive protein, along with purulent discharge observed at the fracture site during surgery and a positive microbial culture result), metaphyseal-epiphyseal non-union, or non-union on a pathological fracture; (2) Concurrent immune disorders, blood system diseases, malignant tumors, diabetes, or other underlying medical conditions; (3) To minimize interference from confounding factors, we opted to include only patients without any pre-existing medical conditions, such as presence of heart, liver, lung, kidney, or another critical organ dysfunction.

Patients were randomized into three groups using a computer-generated random sequence with block randomization (block size = 6) to ensure balanced allocation. Allocation concealment was achieved through sequentially numbered, opaque, sealed envelopes opened by an independent research coordinator after patient enrollment. The surgical team and patients were blinded to group assignment until the intervention was administered.

The surgical procedures were performed by a highly experienced orthopedic surgeon. Initially, a corticocancellous autograft was harvested from the anterior ipsilateral iliac crest. Subsequently, the non-union site was accessed through the original surgical incision. After meticulously removing the excessive scleroproliferative callus and granulation tissue, the fracture ends were fully exposed, and the fracture surface was meticulously polished until fresh bleeding was observed. Following this, all patients were randomized into Groups A, B, and C based on the type of implant utilized. For each patient in Group A, 30 ml of whole blood was drawn from an antecubital vein. The 30 ml syringe was primed with 4 ml of anticoagulant citrate dextrose solution before 26 ml of whole blood was obtained from each patient using standard phlebotomy procedures. On reaching the ﬁnal volume of 30 ml, the syringe was loaded into the Magellan System. The Magellan was programmed to produce 3 ml of PRP from the 30 ml volume. Then, augmentation plating was placed while retaining the original internal fixation, and bone grafting was carried out using a mixture of PRPand autogenous iliac bone. Group B received augmentation plating along with autogenous iliac bone, while Group C was implanted with only autogenous iliac bone.

Preoperatively, an anterior-posterior and lateral radiograph was conducted for each patient. Postoperative radiograph data were gathered at 3, 6, and 9 months, with additional follow-ups conducted as necessary until fracture healing occurred. The callus formation in the x-ray images obtained at 3, 6, and 9 months postoperatively was assessed using the Fernadez-Esteve evaluation criteria as follows (13): Grade I, no radiological callus at the fracture end (0 points); Grade II, cloud callus at the fracture end (1 point); Grade III, callus formation on one side of the fracture in the ortholateral film (2 points); Grade IV, callus formation on both sides of the fracture (3 points); and Grade V, structural callus formation (4 points). Any complications encountered at each time point were meticulously documented.

Statistical analysis was performed using SPSS v19 (IBM, Armonk, New York, USA). Continuous variables are shown as mean ± SD. One-way analysis of variance (ANOVA) was used to determine differences among the respective groups. If the analysis of variance (ANOVA) reveals statistically significant differences (*P* < 0.05), Fisher's least significant difference (LSD) *post hoc* test will be employed to conduct pairwise comparisons between group means. Repeated-measures ANOVA was used to determine differences in the FE scores among the 3 groups. Fisher exact test was used to compare the frequency of events between groups. *P* < 0.05 was considered statistically significant.

## Results

Group A, B and C contained 15 patients, respectively. No statistically significant differences were observed among the three groups in terms of the male-to-female ratio, age, or BMI (Table 1). A comparison of surgical duration, blood loss, length of stay, and fracture healing time among the groups is presented in Table 1. No significant differences in operation time were found between Groups A and B, but significant differences were observed when comparing Groups A and C, as well as Groups B and C. The blood loss in Group A was significantly higher than that in Groups B and C, with no significant difference between Groups B and C. There were no significant differences in length of stay between Groups A and B, or between Groups B and C, but a significant difference was noted between Groups A and C. The fracture healing times among the three groups were statistically different, with Group A having the shortest bone healing time, followed by Group B, and then Group C.

**Table 1:** Demographic and surgical data.

Parameter	Group A	Group B	Group C	*P* Value		
Gender ratio^^^	8:7	9:6	8:7	0.914*		
Age (years)	44.93 ± 7.72	47.07 ± 7.91	46.13 ± 6.86	0.74^#^		
BMI (kg/m^2^)	24.15 ± 2.28	24.86 ± 2.06	23.72 ± 1.95	0.337^#^		
SD (minutes)	124.40 ± 21.83	111.93 ± 19.26	94.47 ± 15.09	0.078^&^	<0.05^$^	0.015^%^
BL (ml)	156.53 ± 32.65	132.27 ± 26.02	114.67 ± 32.04	0.034^&^	<0.05^$^	0.120^%^
LoS (d)	15.33 ± 1.80	14.87 ± 1.96	13.53 ± 2.13	0.520^&^	0.016^$^	0.071^%^
FHT (d)	214.00 ± 45.21	253.40 ± 40.58	374.80 ± 61.08	0.036^&^	<0.05^$^	<0.05^%^

^^^ Male-to-famale ratio.

* One-way analysis of variance.

^#^ Fisher exact test.

^&^ Group A vs. Group B.

^$^ Group A vs. Group C.

^%^ Group B vs. Group C.

Values are mean ± SD or as otherwise indicated.

SD, surgery duration; BL, blood loss; LoS, length of stay; FHT, fracture healing time.

**Figure 1:**
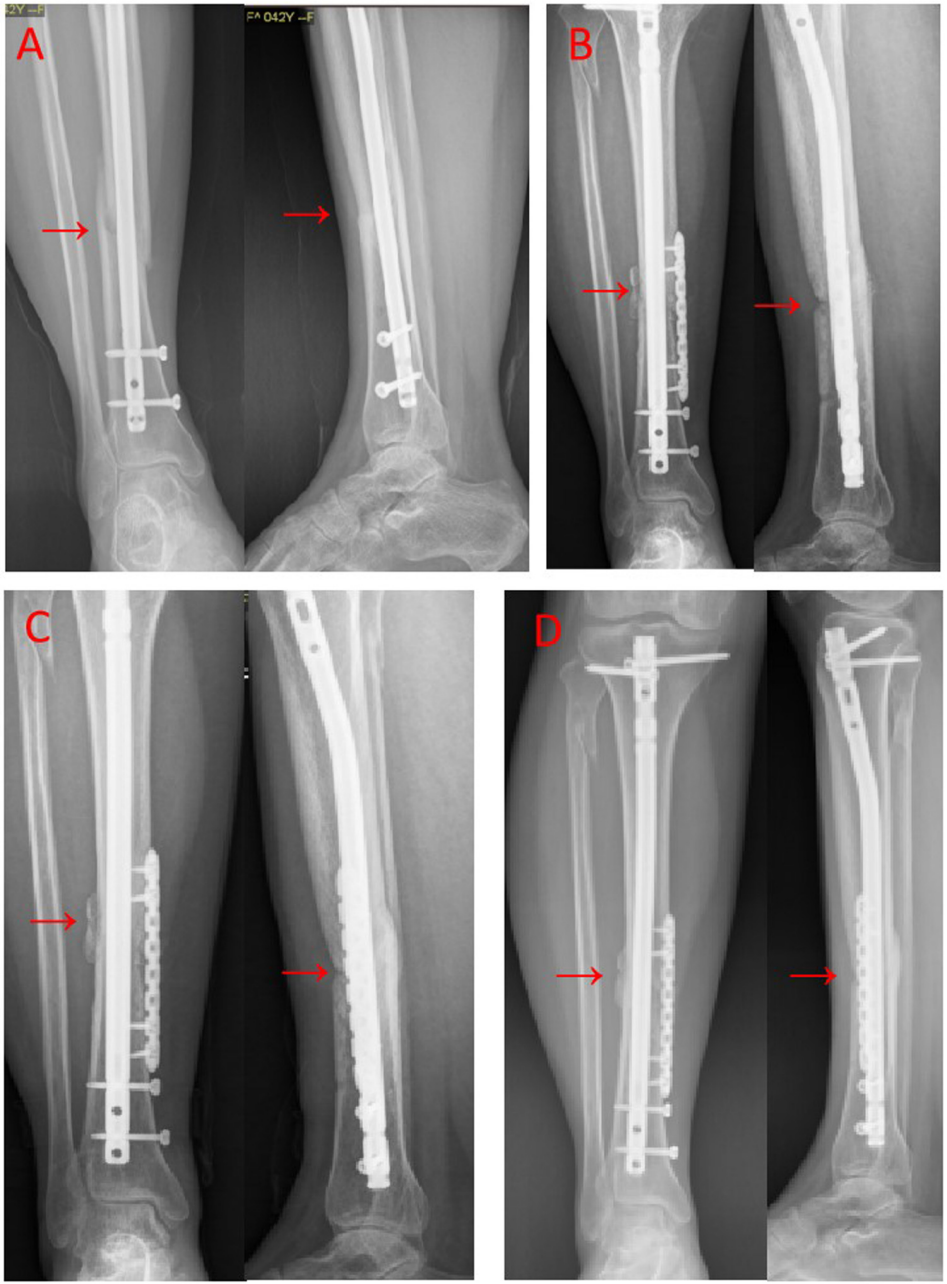
**(A)** After 9 months following closed reduction and intramedullary nail fixation of the fracture in the middle of the right tibia, anterior-lateral radiographs revealed nonunion. **(B)** After 3 months of PRP, augmentation plating and autogenous iliac bone implantation, the anterior-lateral x-ray images demonstrated favorable alignment with a slight increase in callus formation and a hazy appearance. **(C)** 6 months after revision, anterior-lateral radiographs exhibited enhanced callus formation and increased callus on both sides of the fracture. **(D)** 9 months after revision, anterior-lateral x-rays revealed indistinct fracture lines and fully healed fracture ends. The location of the fracture line is clearly indicated by a red arrow.

Table 2 presents a comparison of the postoperative Fernadez-Esteve scores among the three groups. The Fernadez-Esteve scores of all three groups increased significantly over time. At three different time points, Group A exhibited significantly higher Fernadez-Esteve scores compared to Group B, which in turn had higher scores than Group C. A typical case is illustrated in Figure 1.

**Table 2:** Postoperative fernadez-esteve score.

Parameter		Group A	Group B	Group C	*P* Value^#^	*P* Value^$^	*P* Value^%^
Time point	3 months	2.13 ± 0.52	1.67 ± 0.49	1.27 ± 0.46	0.012	<0.05	0.030
	6 months	2.93 ± 0.80	2.40 ± 0.51	1.93 ± 0.46	0.021	0.001	0.041
	9 months	3.87 ± 0.35	3.53 ± 0.83	2.67 ± 0.49	0.132	<0.05	<0.05
*P* Value^^^		0.003	<0.05	<0.05			
*P* Value^&^		<0.05	<0.05	<0.05			
*P* Value*		<0.05	<0.05	<0.05			

^#^ Group A vs. Group B.

^$^ Group A vs. Group C.

^%^ Group B vs. Group C.

^^^ Group 3 months vs. 6 months.

^&^ Group 6 months vs. 9 months.

* 6 months vs. 9 months.

Values are mean ± SD or as otherwise indicated.

## Discussion

Nonunion denotes the failure of fracture ends to achieve satisfactory healing within a period of 6 to 9 months post-fracture surgery, accompanied by an absence of any discernible increase in bone callus after an additional three months of continuous monitoring (14, 15). In China, the occurrence rate of nonunion typically falls between 2% and 7%, with tibial nonunion constituting a significant proportion of 45% to 65% among these cases (16). This is predominantly attributed to the tibial medial soft tissue's limited blood supply and susceptibility to infection, rendering it a prevalent site for nonunion development. Extensive research is currently being conducted on clinical treatments for fracture nonunion, encompassing autologous bone grafting, gene therapy, membrane-guided regeneration, and bone tissue engineering techniques (17). These methodologies embody diverse therapeutic strategies, all aimed at enhancing the efficiency and success rate of fracture healing.

Bone grafting is widely recognized as an effective treatment for nonunion. The most common approach in clinical practice is the use of autologous bone grafting, as it directly derives from the patient's own body and has good tissue compatibility, with no immune rejection reactions after transplantation (18). Additionally, autologous bone grafting can effectively promote fracture healing and regeneration, making it the gold standard for bone fusion in orthopedic surgery (19). While allogenic bone grafting helps solve the problem of limited bone supply and scope in autologous bone grafting, thereby shortening the time required for surgery, it has a relatively higher failure rate due to the risk of rejection, and the patient's treatment cost is also high (20). Research has shown that autologous bone grafting for nonunion can reduce the risk of infection and improve healing rates, playing an important role in clinical practice (21).

The application of augmentation plating has brought forth new therapeutic prospects for postoperative fracture nonunion. On the condition that the original internal fixation devices remain stable and not loosened, the approach assisted by augmentation plating can make up for the deficiency of poor rotational stability of the affected limb (22). Meanwhile, this method enhances the axial stability of the affected limb and provides a stable biomechanical environment for the calcification of local fibrocartilage and the formation of callus (23). Research has shown that the treatment of tibial bone nonunion with augmentation plating can reduce intraoperative hemorrhage, increase the stability of the fracture ends, improve the joint mobility of the affected limb, and is highly safe (24). Ye, J et al. have demonstrated through experiments that augmentation plating possesses the merits of straightforward operation, minor trauma, and favorable therapeutic efficacy, and can be employed in the treatment of postoperative long bone nonunion (25).

The advent of PRP technology undoubtedly offers novel therapeutic choices for patients with nonunion of bones. The mechanism by which PRP promotes the repair of nonunion of bones mainly concentrates on three aspects, namely inflammatory cytokines, growth factors, and angiogenic factors. The therapeutic efficacy of PRP in bone healing primarily stems from its rich reservoir of growth factors, including platelet-derived growth factor (PDGF), transforming growth factor-beta (TGF-β), vascular endothelial growth factor (VEGF), and insulin-like growth factor (IGF). These factors synergistically activate critical signaling pathways involved in osteogenesis and angiogenesis. For instance, TGF-β promotes mesenchymal stem cell (MSC) differentiation into osteoblasts via the SMAD pathway (26). VEGF enhances vascularization by stimulating endothelial cell proliferation through the PI3K/Akt pathway (27). PDGF recruits osteoprogenitor cells to the fracture site and upregulates extracellular matrix synthesis (28). Recent preclinical studies further demonstrate that PRP-derived exosomes modulate the Wnt/β-catenin pathway, enhancing bone regeneration by promoting osteoblast proliferation and suppressing osteoclast activity. Currently, in the treatment of nonunion of bones, as a catalyst in combination with transplanted bone for treating nonunion, it promotes the regeneration of connective tissue and bones. Platelets themselves contain interleukin, and activated platelets can express IL-1 and IL-6 (29). Interleukin plays a crucial role in initiating bone repair, chemotaxis of monocytes and macrophages, and stimulation of angiogenesis (30). The growth factors secreted by PRP can generate activity under the effect of histone and carbohydrate side chains, combine with osteoblasts, mesenchymal stem cells, etc., trigger a series of intracellular signal transduction, and accelerate biological processes such as cell proliferation, matrix formation, and cell differentiation (31). Roffi contends that through multi-data analysis, it is concluded that PRP has significant clinical efficacy and high safety in the treatment of nonunion of bones (32). This research provides strong support for the application of PRP in the field of fracture healing, emphasizing it as an effective and safe therapeutic option (33).

The present study demonstrated that the combination of PRP with autologous bone graft and augmentation plate in the treatment of tibia nonunion resulted in significantly improved fracture healing time and x-ray callus score at different time points compared to the another groups during the same period, indicating clear clinical efficacy. In this particular study, the mean duration for bony healing was observed to be 214 days in group A, 253 days in group B, and 374 days in group C. Notably, expedited bone healing enables patients to resume walking unaided by crutches at an earlier stage, thereby enhancing their overall quality of life. Additionally, this accelerated healing process contributes to a lower likelihood of requiring re—operation. However, it is worth noting that this study has limitations due to a relatively small sample size. In future research, multi-center and large-sample trials should be conducted to provide more comprehensive theoretical and data support for the clinical management of bone nonunion.

## Conclusion

The clinical efficacy and safety of combined used of PRP, augmentation plating and autogenous bone in patients with nonunion of tibia were satisfied. However, the sample size of this research was relatively small, and additional studies with longer follow-up periods are needed to demonstrate the clinical effectiveness.

## Data Availability

The datasets presented in this article are not readily available because The data are available from the corresponding author on reasonable request. Requests to access the datasets should be directed to 18826401776@163.com.
