# Supplementary material for: Clinical outcomes of tibial nonunion treatment through the combination of PRP, autogenous iliac bone grafting, and augmentation plating
Source: Front Surg. 2025 Jul 3;12:1573679. doi: 10.3389/fsurg.2025.1573679 (PMC12267238; doi:10.3389/fsurg.2025.1573679)
Supplement: Supplementary file 1 [file Table1.docx]

Table 1. Demographic Data

| Parameter | Group A(n=15) | Group B(n=15) | Group C(n=15) | *P* Value |
| --- | --- | --- | --- | --- |
| Male-to-female ratio | 8:7 | 9:6 | 8:7 | 0.914* |
| Age (years) | 44.93±7.72 | 47.07±7.91 | 46.13±6.86 | 0.74^#^ |
| BMI (kg/m^2^) | 24.15±2.28 | 24.86±2.06 | 23.72±1.95 | 0.337^#^ |

Values are mean±SD or as otherwise indicated.

Group A, Group B, Group C

*One-way analysis of variance.

^#^Fisher exact test.

Table 2. Surgical and Clinical Data

| Parameter | Group A | Group B | Group C | *P* Value^#^ | *P* Value^$^ | *P* Value^%^ |
| --- | --- | --- | --- | --- | --- | --- |
| SD(minutes) | 124.40±21.83 | 111.93±19.26 | 94.47±15.09 | 0.078 | ＜0.05 | 0.015 |
| BL (mL) | 156.53±32.65 | 132.27±26.02 | 114.67±32.04 | 0.034 | ＜0.05 | 0.120 |
| LoS (d) | 15.33±1.80 | 14.87±1.96 | 13.53±2.13 | 0.520 | 0.016 | 0.071 |
| FHT(d) | 214.00±45.21 | 253.40±40.58 | 374.80±61.08 | 0.036 | ＜0.05 | ＜0.05 |

Values are mean±SD or as otherwise indicated.

SD, surgery duration; BL, blood loss; LoS, length of stay; FHT, fracture healing time.

^#^Group A vs Group B, ^$^Group A vs Group C, ^%^Group B vs Group C.

Table 3. Postoperative Fernadez-esteve score

| Parameter | | Group A | Group B | Group C | *P* Value^#^ | *P* Value^$^ | *P* Value^%^ |
| --- | --- | --- | --- | --- | --- | --- | --- |
| Time point | 3 months | 2.13±0.52 | 1.67±0.49 | 1.27±0.46 | 0.012 | ＜0.05 | 0.030 |
|  | 6 months | 2.93±0.80 | 2.40±0.51 | 1.93±0.46 | 0.021 | 0.001 | 0.041 |
|  | 9 months | 3.87±0.35 | 3.53±0.83 | 2.67±0.49 | 0.132 | ＜0.05 | ＜0.05 |
| *P* Value^ | | 0.003 | ＜0.05 | ＜0.05 |  |  |  |
| *P* Value^&^ | | ＜0.05 | ＜0.05 | ＜0.05 |  |  |  |
| *P* Value* | | ＜0.05 | ＜0.05 | ＜0.05 |  |  |  |

Values are mean±SD or as otherwise indicated.

^#^Group A vs Group B, ^$^Group A vs Group C, ^%^Group B vs Group C.

^Group 3 months vs 6 months, ^&^Group 6 months vs 9 months, *6 months vs 9 months.
